# Common concepts for bacterial collectives

**DOI:** 10.7554/eLife.47019

**Published:** 2019-04-30

**Authors:** Hannah Jeckel, Noémie Matthey, Knut Drescher

**Affiliations:** 1 Max Planck Institute for Terrestrial MicrobiologyMarburgGermany; 2 Department of PhysicsPhilipps-Universität MarburgMarburgGermany; 3 School of Life SciencesSwiss Federal Institute of Technology LausanneLausanneSwitzerland

**Keywords:** biofilms, collective spreading, biophysics, *B. subtilis*

## Abstract

The expansion of bacterial swarms and the spreading of biofilms can be described by a unified biophysical theory that involves both active and passive processes.

**Related research article** Srinivasan S, Kaplan CN, Mahadevan L. 2019. A multiphase theory for spreading microbial swarms and films. *eLife* **8**:e42697. doi: 10.7554/eLife.42697

A long-held paradigm in microbiology has been that bacteria are unicellular creatures, and that the effect of a large population of bacteria is the sum of the effects of all the individual cells. However, this view of bacterial life has changed fundamentally over the last few decades (Parsek and Greenberg, 2005), and it is now clear that bacteria can communicate with each other via small molecules and coordinate their behavior. Bacteria can, for example, form swarms as they move across a surface to explore their surroundings and search for nutrients, and they can also grow into multicellular structured communities termed biofilms, which provide various benefits to the cells, such as an improved tolerance to antibiotics. The study of behaviors that only occur in groups of bacterial cells now represents an exciting frontier in microbiology research. It has also caught the attention of physicists and mathematicians who are interested in identifying the quantitative principles that underpin multicellular organization.

Bacterial swarms and biofilms are typically viewed as two fundamentally different phenotypes because they are regulated by different genes, are caused by different cellular processes, and display obvious differences at the microscopic scale: swarms involve highly motile cells that move collectively (Darnton et al., 2010; Kearns, 2010; Zhang et al., 2010), whereas biofilms consist primarily of non-motile cells that are held together by an extracellular matrix (Flemming et al., 2016). However, at a more macroscopic level of description, there are also some similarities: swarms and biofilms both involve the spreading of bacterial communities with low height-to-width ratios over an agar substrate, and both consist of multiple different components (cells, matrix and fluid).

**Figure 1.**
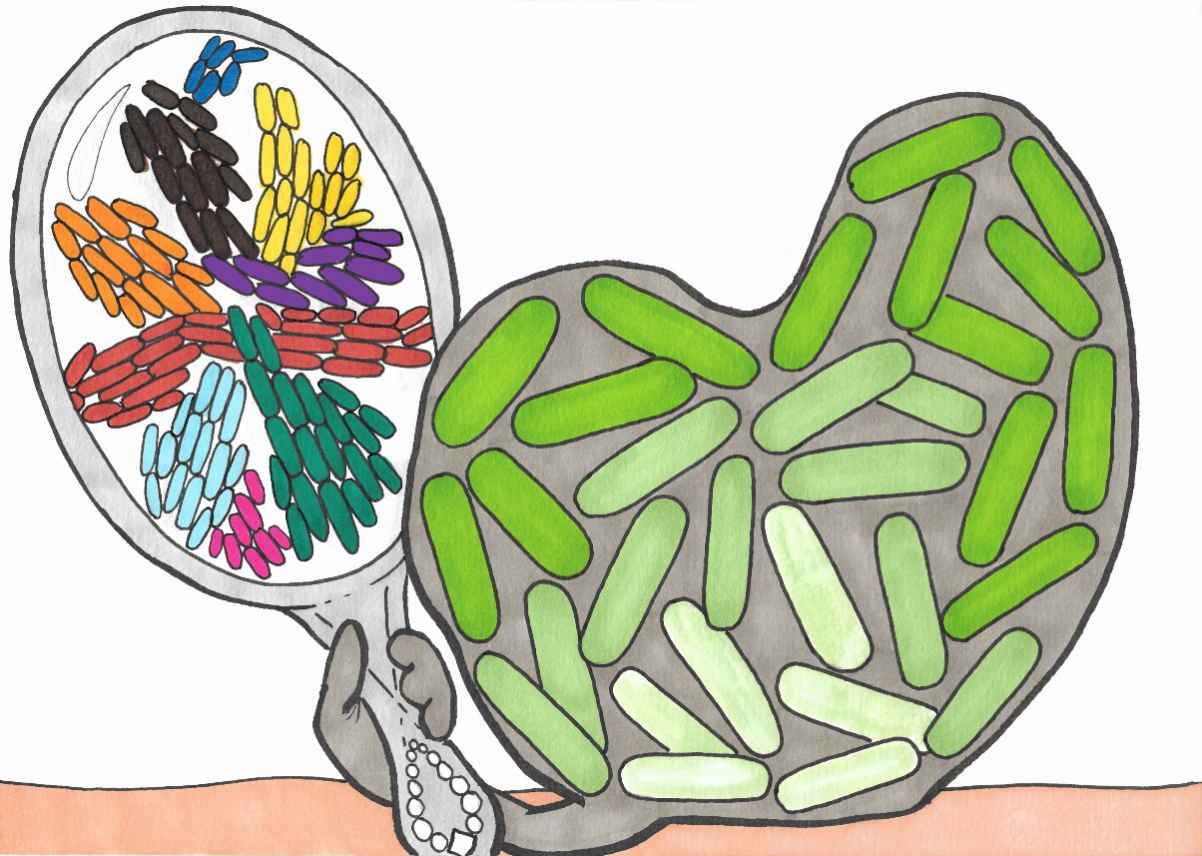
Biofilms and swarms are similar in many ways. Illustration showing a biofilm (right) made of bacteria (green shapes) and extracellular matrix (gray) looking at itself in a mirror and seeing a swarm of bacteria, where different colors indicate groups of cells that are moving together in the same direction. Srinivasan et al. have shown that the spreading dynamics of swarms and biofilms at the macroscopic level can be described by a single biophysical theory. Illustration by Noémie Matthey.

Now, in *eLife*, inspired by these similarities, Siddarth Srinivasan, Nadir Kaplan and L Mahadevan of Harvard University report that they have developed a unifying theory that can describe the expansion of both swarms and biofilms (Srinivasan et al., 2019). Srinivasan *et al.* realized that the spread of both systems can be viewed as involving just two phases of matter: an active, growing phase of bacteria and/or matrix components, and a passive phase of fluid which can move from the substrate to the system and back. Based on this insight, Srinivasan *et al.* formulated a two-phase model which establishes a general set of coupled equations that include the effects of growth, fluid fluxes, nutrients and diffusion, with the specific components of the equations being adapted for swarms and biofilms. They found that despite the differences between swarms and biofilms, their model could quantitatively predict the dynamics of how they expand.

The model neglects biological details such as gene regulation and the actual mechanisms of cell-cell interactions. Instead, it relies only on growth and physical effects. A remarkable conclusion of the fact that such a theory quantitatively reproduces the bacterial spreading dynamics is that, on the macroscopic scale, physical interactions between the two phases and the agar substrate together with cellular growth are the driving factors of colony expansion.

Similarly, studies focusing on the microscopic dynamics of swarms and biofilms have identified a unifying concept for cell-cell interactions: physical interactions (such as collisions between cells) determine both the architecture of a biofilm and the collective movement of a swarm (Hartmann et al., 2019; Jeckel et al., 2019; Trejo et al., 2013; Wensink et al., 2012). This suggests that while biological mechanisms determine the physiological state of cells and the type of community they build, physical interactions can in certain conditions predict the structure of this community and the dynamics of the cells within the community both microscopically and macroscopically.

Taken together with previous work on the similarities between swarms and biofilms at microscopic length scales (Hartmann et al., 2019; Jeckel et al., 2019), the results of Srinivasan *et al.* are another step forward in efforts to identify general principles that are able to explain the behavior of bacterial communities across a range of length scales and across species.
